# Least-Squares Fitting Algorithms of the NIST Algorithm Testing System

**DOI:** 10.6028/jres.103.043

**Published:** 1998-12-01

**Authors:** Craig M. Shakarji

**Affiliations:** National Institute of Standards and Technology, Gaithersburg, MD 20899-0001

**Keywords:** coordinate measuring machine, curve fitting, least-squares fitting, Levenberg-Marquardt, orthogonal distance regression, surface fitting

## Abstract

This report describes algorithms for fitting certain curves and surfaces to points in three dimensions. All fits are based on orthogonal distance regression. The algorithms were developed as reference software for the National Institute of Standards and Technology’s Algorithm Testing System, which has been used for 5 years by NIST and by members of the American Society of Mechanical Engineers’ B89.4.10 standards committee. The Algorithm Testing System itself is described only briefly; the main part of this paper covers the general linear algebra, numerical analysis, and optimization methods it employs. Most of the fitting routines rely on the Levenberg-Marquardt optimization routine.

## 1. Introduction

Mathematical software, particularly curve and surface fitting routines, is a critical component of coordinate metrology systems. An important but difficult task is to assess the performance of such routines [1,2]. The National Institute of Standards and Technology has developed a software package, the NIST Algorithm Testing System (ATS), that can assist in assessing the performance of geometry-fitting routines [3]. This system has been aiding in the development of a U. S. standard (American Society of Mechanical Engineers (ASME) B89.4.10) for software performance evaluation of coordinate measuring systems [4]. The ATS is also the basis of NIST’s Algorithm Testing and Evaluation Program for Coordinate Measuring Systems (ATEP-CMS) [5,6].

The ATS incorporates three core modules: a *data generator* [7] for defining and generating test data sets, a collection of *reference algorithms* which provides a performance baseline for fitting algorithms, and a *comparator* for analyzing the results of fitting algorithms versus the reference algorithms. This paper concentrates on the development of highly accurate reference algorithms.

The paper is organized as follows. We first introduce notation and certain key functions and derivatives that will be used throughout the paper. Section 2 describes fitting algorithms for linear geometries—planes and lines. Lagrange multipliers [8] are used in solving the constrained minimization problems, which are developed into standard eigenvector problems. Section 3 deals with nonlinear geometry fitting. We use an unconstrained optimization method that requires derivatives of appropriate distance functions. These required functions and derivatives are provided for the reader. Appendix A gives an outline of the unconstrained optimization algorithm (Levenberg-Marquardt) that is modified to allow for normalization of fitting parameters within the routine. Appendix B gives, for all the geometries, the appropriate derivatives needed to create a valuable check for a local minimum.

### 1.1 Notation and Preliminary Remarks

Assume we are fitting a set of data points, {***x****_i_*}, *i* = 1,2,…, *N*, that have been translated so that their centroid is the origin. Usually scalar quantities are represented in plain type and matrix or vector quantities with boldface type. Other notation:

**Table:** 

***x*** = (*x*,*y*,*z*)	A point in 3-dimensional space.
|·|	The Euclidean (***L***_2_) norm. E. g., $\left|x\right|=\sqrt{x^{2}+y^{2}+z^{2}}$.
***x****_i_* = (*x_i_*,*y_i_*,z_i_)	The *i*th data point.
$\bar{x}=(\bar{x},\bar{y},\bar{z})$	The centroid of the data, $\frac{1}{N}\left(\sum x_{i},\sum y_{i},\sum z_{i}\right)$ (Note: These and all other sums in this paper are taken from *i* = 1,2,…,*N*.)
***A*** = (*A*,*B*,*C*)	Direction numbers that specify an orientation, ***A*** ≠ 0.
***a*** = (*a*,*b*,*c*)	Direction cosines that specify an orientation. Note: |***a***| = 1. An orientation’s direction numbers can be converted into direction cosines by: ***a*** = ***A***/|***A***|.
*J*	The objective function. *J* is the sum of the squares of the distances from the data points to the geometry. $J=\sum d_{i}^{2}$.
***M***	The *N* × 3 matrix containing the data points: $\left[\begin{array}{ccc} x_{1} & y_{1} & z_{1}\\ x_{2} & y_{2} & z_{2}\\ \vdots & \vdots & \vdots \\ x_{N} & y_{N} & z_{N} \end{array}\right]$
∇	The gradient of a scalar function. E.g., $\nabla h(x,y,z)=(h_{x},h_{y},h_{z})=\left(\frac{\partial h}{\partial x},\frac{\partial h}{\partial y},\frac{\partial h}{\partial z}\right)$.

For each geometry, we show the defining parameters, the equation for the orthogonal distance from a single data point to the geometry, the objective function, and a brief description of the steps in the calculation.

## 2. Linear Geometries

Linear geometries (lines and planes) are solved using Lagrange multipliers on a constrained minimization problem. Both cases reduce to a rather simple eigen-problem.

### 2.1 Plane Fitting

Defining parameters:

***x***—a point on the plane.
***a***—the direction cosines of the normal to the plane.

Distance equation:

$$d_{i}=d(x_{i})=d(x_{i},x,a)=a\cdot (x_{i}-x)$$

Objective function:

$$J(x,a)={\sum \left[a\cdot (x_{i}-x)\right]}^{2}$$

Description:

The centroid of the data must lie on the least-squares plane. This can be seen because ∇*J* = 0 at the least squares solution, yielding $\sum a\cdot (x_{i}-x)=0$. Multiplying by 1/*N* gives $\frac{a}{N}\sum (x_{i}-x)+\frac{b}{N}\sum \left(y_{i}-y\right)+\frac{c}{N}\sum \left(z_{i}-z\right)=0$. Distributing the summation gives $a(\bar{x}-x)+b(\bar{y}-y)+c(\bar{z}-z)=0$, which is to say $d(\bar{x},x,A)=0$, i.e., $\bar{x}$ lies on the least-squares plane. Since by assumption the data points have been translated to the origin, and since the centroid of the data must be a point on the least squares plane, we can set ***x*** = 0.

The direction of the fitted plane, ***a***, can be found by solving the constrained minimization problem, namely, minimizing *J* subject to the constraint that |***a***| = 1. Define a function, *G* = |***a***|^2^ − 1, so that the problem is to minimize *J* subject to the constraint that *G* = 0. The method of Lagrange multipliers [8] tells us that the minimum occurs at a point where ∇*J* = λ∇*G*, for some real number λ. (Here, *a*, *b*, and *c* are treated as independent variables, since the constraint is accounted for in *G*. Therefore, ∇ = (∂/∂*a*, ∂/∂*b*, ∂/∂*c*).) But ∇*G* = 2***a***, and ∇*J* = 2(***M***^⊤^***M***)***a***, yielding the eigen-problem, (***M***^⊤^***M***)***a*** = λ***a***, referred to as the *normal equations*.

This 3 × 3 eigenvector problem can be easily solved using well-established routines (e.g., Jacobi iterations [9]). However, we note that the eigenvectors of ***M***^⊤^***M*** are also the singular vectors (from the singular value decomposition) of ***M*** [9]. This allows us to gain numerical stability by applying the singular value decomposition (SVD) to ***M*** without ever computing ***M***^⊤^***M***, which is the method implemented in the ATS.

Finally, we must determine how to select the correct eigenvector (i.e., singular vector) of the three produced by the SVD. The normal equations can be written as follows:

$$\begin{array}{l} \Sigma \;x_{i}(a\cdot x_{i})=\lambda a\\ \Sigma \;y_{i}(a\cdot x_{i})=\lambda b\\ \Sigma \;z_{i}(a\cdot x_{i})=\lambda c \end{array}$$

Multiplying these three equations by *a*, *b*, and *c*, respectively, then summing the equations gives $\sum {\left(a\cdot x_{i}\right)}^{2}=\lambda {\left|a\right|}^{2}=\lambda$.

But the sum on the left is just the objective function, i.e., the sum of the squares of the residuals. Thus the value of λ is the objective function, hence the correct eigenvector for the least-squares solution corresponds to the smallest eigenvalue. When using the SVD, we choose the singular vector corresponding to the smallest singular value, since under these conditions the singular values are the square roots of the eigenvalues [10].

### 2.2 Line Fitting

Defining parameters:

***x***—a point on the line.
***a***—the direction cosines of the line.

Distance equation:

*d*(***x****i*) = *d*(***x****i*, ***x***,***a***) = |***a*** × (***x****i* − ***x***)| (which also equals $\sqrt{{\left|x_{i}-x\right|}^{2}-{\left[a\cdot (x_{i}-x)\right]}^{2}}$ where × denotes the vector cross product.

Objective function:

$$J(x,a)=\sum {\left|a\times (x_{i}-x)\right|}^{2}$$

Description:

Since the data has been translated to the origin, and since the centroid of the data must be a point on the least squares plane (similar to the case above with the least squares plane), we set ***x*** = 0.

The direction ***a*** can be found by following the same strategy as in the case of a plane. For line fitting, the normal equations are ***M***^⊤^***Ma*** = λ***a*** just as in the case of plane fitting. Once again, the correct eigenvector must be chosen to minimize the sum-of-squares of the residuals. As shown with planes, we obtain λ = Σ(***a***·***x***)^2^, so *J* = − λ + ∑|***x****_i_*|^2^, meaning that *J* is minimized when λ is maximized. Thus the correct eigenvector choice is the one corresponding to the largest eigenvalue. As in the case of plane fitting, numerical stability is gained by finding the eigenvectors of ***M*** through the SVD, rather than by solving the normal equations. Since the singular values are the square roots of the eigenvalues [10], we choose the eigenvector corresponding to the largest singular value.

## 3. Nonlinear Geometries

### 3.1 Utility Functions *f* and *g*

The line and plane distance functions arise quite often in this paper, thus we define them here, calling them *f* and *g* respectively, giving necessary derivatives, which are used throughout the rest of this paper. We compute the nonlinear fits using unconstrained minimization algorithms, so we define the line and plane distance functions in terms of direction numbers rather than direction cosines.

Let *g*(***x****_i_*,***x***,***A***) denote the distance from the point, ***x****_i_*, to the plane defined by the point, ***x***, and the normal direction, ***a*** = ***A***/|***A***|. The value of *g* is given by: *g_i_* = *g*(***x****_i_*,***x***,***A***) = ***a***·(***x****_i_* − ***x***) = *a*(*x_i_* − *x*) + *b*(*y_i_* − *y*) + *c*(*z_i_* − *z*).

Let *f*(***x****_i_*,***x***,***A***) denote the distance from the point, ***x****_i_*, to the line defined by the point, ***x***, and the direction, ***a*** = ***A***/|***A***|. The value of *f* is given by: *f_i_* = *f*(***x****_i_*,***x***,***A***) = |***a*** × (***x****_i_* − ***x***)|. That is,

$$\begin{array}{r} \;u=c(y_{i}-y)-b(z_{i}-z)\\ f_{i}=\sqrt{u^{2}+v^{2}+w^{2}},\;\text{where}\;v=a(z_{i}-z)-c(x_{i}-x)\\ w=b(x_{i}-x)-a(y_{i}-y) \end{array}$$

This expression for *f* is used because of its numerical stability. One should note that *f* could also be expressed (for derivative calculations) as $f_{i}=\sqrt{{\left|x_{i}-x\right|}^{2}-g_{i}^{2}}$.

Note: *A*, *B*, and *C* are independent variables, whereas *a*, *b*, and *c* are not, because the constraint *a*^2^ + *b*^2^ + *c*^2^ = 1 causes *a*, *b*, and *c* to depend on each other. When dealing with the nonlinear geometries we treat *f* and *g* as functions dependent on ***A***, as opposed to ***a***, in order to use unconstrained minimization algorithms. Treating *f* and *g* as functions dependent on ***a*** would force us to restrict ourselves to using constrained minimization solvers. In the linear cases, we *did* solve constrained minimization problems. So when we differentiate with respect to *A*, for example, we treat *a*, *b*, and *c* all as functions of *A*, *B*, and C (e.g., $a=A/\sqrt{A^{2}+B^{2}+C^{2}})$. This yields the following array of derivatives:

$$\left[\begin{array}{c} \nabla a\\ \nabla b\\ \nabla c \end{array}\right]=\left[\begin{array}{ccc} \frac{\partial a}{\partial A} & \frac{\partial a}{\partial B} & \frac{\partial a}{\partial C}\\ \frac{\partial b}{\partial A} & \frac{\partial b}{\partial B} & \frac{\partial b}{\partial C}\\ \frac{\partial c}{\partial A} & \frac{\partial c}{\partial B} & \frac{\partial c}{\partial C} \end{array}\right]=\frac{1}{\left|A\right|}\left[\begin{array}{lll} 1-a^{2} & -ab & -ac\\ -ab & 1-b^{2} & -bc\\ -ac & -bc & 1-c^{2} \end{array}\right]$$

These algorithms normalize ***A*** at every step, so for simplicity of expressing derivatives, assume |***A***| = 1. ***A*** remains unconstrained; we just assume it happens to have unit magnitude.

The derivatives for *f_i_* and *g_i_* are then

$$\begin{array}{l} \frac{\partial g_{i}}{\partial x}=-a\\ \frac{\partial g_{i}}{\partial y}=-b\\ \frac{\partial g_{i}}{\partial z}=-c\\ \frac{\partial g_{i}}{\partial A}=(x_{i}-x)-ag_{i}\\ \frac{\partial g_{i}}{\partial B}=(y_{i}-y)-bg_{i}\\ \frac{\partial g_{i}}{\partial C}=(z_{i}-z)-cg_{i}\\ \frac{\partial f_{i}}{\partial x}=[ag_{i}-(x_{i}-x)]/f_{i}\\ \frac{\partial f_{i}}{\partial y}=[bg_{i}-(y_{i}-y)]/f_{i}\\ \frac{\partial f_{i}}{\partial z}=[cg_{i}-(z_{i}-z)]/f_{i}\\ \frac{\partial f_{i}}{\partial A}=g_{i}[ag_{i}-(x_{i}-x)]/f_{i}\\ \frac{\partial f_{i}}{\partial B}=g_{i}[bg_{i}-(y_{i}-y)]/f_{i}\\ \frac{\partial f_{i}}{\partial C}=g_{i}[cg_{i}-(z_{i}-z)]/f_{i} \end{array}$$

The above derivatives of *f_i_* are undefined when *f_i_* = 0 (i.e., when ***x****_i_* is on the line.) In this rarely needed case the gradient is given by:

$$\begin{array}{l} (\sqrt{1-a^{2}},\sqrt{1-b^{2}},\sqrt{1-c^{2}},\\ g\sqrt{1-a^{2}},g\sqrt{1-b^{2}},g\sqrt{1-c^{2}}). \end{array}$$

For cylinders, cones, and tori, the line associated with *f* is the geometry’s axis. For cones the plane associated with *g* is the plane through the point, ***x***, perpendicular to the axis. For tori, the plane associated with ***g*** is the plane perpendicular to the axis that divides the torus in half.

### 3.2 Choice of Algorithm

Good optimization algorithms can readily be found to minimize the objective function, *J*. Usually such an algorithm will require an initial guess, along with partial derivatives, either of *J* itself or of the distance function, *d_i_*. Both sets of derivatives are given in this paper (those for *J* in the appendix). These should enable a reader to implement a least-squares algorithm even if the optimization algorithm used differs from the author’s choice, which follows.

In the ATS, nonlinear geometries are fit in an unconstrained manner using the Levenberg-Marquardt algorithm. The algorithm requires an initial guess as well as the first derivatives of the distance function. In practice it converges quickly and accurately even with a wide range of initial guesses. Details of this algorithm are given in appendix A. Additionally, the code allows us to normalize the fitting parameters after every iteration of the algorithm. For each geometry we list the distance and objective functions, the appropriate derivatives, and the parameter normalization we use.

### 3.3 Sphere Fitting

Defining parameters:

***x***—the center of the sphere.
*r*—the radius of the sphere.

Distance equation:

$$d(x_{i})=\left|x_{i}-x\right|-r$$

Objective function:

$$J(x_{i}r)=\sum {\left(|x_{i}-x|-r\right)}^{2}$$

Normalization:

(None)

Derivatives:

$$\begin{array}{l} \frac{\partial d_{i}}{\partial x}=-(x_{i}-x)/|x_{i}-x|\\ \frac{\partial d_{i}}{\partial y}=-(y_{i}-y)/|x_{i}-x|\\ \frac{\partial d_{i}}{\partial z}=-(z_{i}-z)/|x_{i}-x|\\ \frac{\partial d_{i}}{\partial r}=-1 \end{array}$$

### 3.4 Two-Dimensional Circle Fitting

This case is simply the sphere fit (above) restricted to two-dimensions:

Defining parameters:

*x*—the *x*-coordinate of the center of the circle.
*y*—the *y*-coordinate of the center of the circle.
*r*—the radius of the circle.

Distance equation:

$$d(x_{i},y_{i})=\sqrt{{\left(x_{i}-x\right)}^{2}+{\left(y_{i}-y\right)}^{2}}-r$$

Objective function:

$$J(x,y,r)=\sum {\left(\sqrt{{\left(x_{i}-x\right)}^{2}+{\left(y_{i}-y\right)}^{2}}-r\right)}^{2}$$

Normalization:

(None)

Derivatives:

$$\begin{array}{l} \frac{\partial d_{i}}{\partial x}=-(x_{i}-x)/(d_{i}+r)\\ \frac{\partial d_{i}}{\partial y}=-(y_{i}-y)/(d_{i}+r)\\ \frac{\partial d_{i}}{\partial r}=-1 \end{array}$$

### 3.5 Three-Dimensional Circle Fitting

Defining parameters:

***x***—the center of the circle.
***A***—the direction numbers of the normal to circle’s plane.
*r*—the radius of the circle.

Distance equation:

$$d(x_{i})=\sqrt{g_{i}^{2}+{\left(f_{i}-r\right)}^{2}}$$

Objective function:

$$J(x,A,r)=\sum \left(g_{i}^{2}+{\left(f_{i}-r\right)}^{2}\right)$$

Normalization:

**Table:** 

***A*** ← ***A***/|***A***|	(Here and elsewhere, “←” denotes assignment of value. In this case, the value of ***A*** is replaced by the value ***A***/|***A***|.)

Derivatives:

$$\begin{array}{l} \frac{\partial d_{i}}{\partial x}=\left[g_{i}{\left(g_{i}\right)}_{x}+f_{i}{\left(f_{i}\right)}_{x}\right]/d_{i}\\ \frac{\partial d_{i}}{\partial y}=\left[g_{i}{\left(g_{i}\right)}_{y}+f_{i}{\left(f_{i}\right)}_{y}\right]/d_{i}\\ \frac{\partial d_{i}}{\partial z}=\left[g_{i}{\left(g_{i}\right)}_{z}+f_{i}{\left(f_{i}\right)}_{z}\right]/d_{i}\\ \frac{\partial d_{i}}{\partial A}=\left[g_{i}{\left(g_{i}\right)}_{A}+f_{i}{\left(f_{i}\right)}_{A}\right]/d_{i}\\ \frac{\partial d_{i}}{\partial B}=\left[g_{i}{\left(g_{i}\right)}_{B}+f_{i}{\left(f_{i}\right)}_{B}\right]/d_{i}\\ \frac{\partial d_{i}}{\partial C}=\left[g_{i}{\left(g_{i}\right)}_{C}+f_{i}{\left(f_{i}\right)}_{C}\right]/d_{i}\\ \frac{\partial d_{i}}{\partial r}=-\left(f_{i}-r\right)/d_{i} \end{array}$$

Description:

We use a multi-step process to accomplish 3D circle fitting:

1. Compute the least-squares plane of the data.
2. Rotate the data such that the least-squares plane is the *x*-*y* plane.
3. Project the rotated data points onto the *x*-*y* plane.
4. Compute the 2D circle fit in the *x*-*y* plane.
5. Rotate back to the original orientation.
6. Perform a full 3D minimization search over all the parameters.

Some coordinate measuring system software packages stop at step (5) and report the orientation, center, and radius as the least-squares circle in 3D. This approach is valid when the projection onto the plane is done simply to compensate for measurement errors on points which would otherwise be coplanar. But this method does not in general produce the circle yielding the least sum-of-squares possible (even though it is usually a good approximation.) In order to achieve the true 3D least-squares fit, the circle computed at step (5) is used as an initial guess in the Levenberg-Marquardt algorithm, which optimizes over all the parameters simultaneously [step (6)].

Step (2) is carried out using the appropriate rotation matrix to rotate the direction, ***a***, to the *z*-direction, namely,

$$\left[\begin{array}{ccc} 1-\frac{a^{2}}{1+c} & \frac{-ab}{1+c} & -a\\ \frac{-ab}{1+c} & 1-\frac{b^{2}}{1+c} & -b\\ a & b & c \end{array}\right]$$

If *c* < 0, ***a*** is replaced with − ***a***, thus rotating the direction to the minus *z*-direction (which is adequate for our purposes.) Step (5) is carried out using the appropriate rotation matrix to rotate the *z*-direction to the direction, ***a***. Namely,

$$\left[\begin{array}{ccc} 1-\frac{a^{2}}{1+c} & \frac{-ab}{1+c} & a\\ \frac{-ab}{1+c} & 1-\frac{b^{2}}{1+c} & b\\ -a & -b & c \end{array}\right]$$

### 3.6 Cylinder Fitting

Defining parameters:

***x***—a point on the cylinder axis.
***A***—the direction numbers of the cylinder axis.
*r*—the radius of the cylinder.

Distance equation:

$$d(x_{i})=f_{i}-r$$

Objective function:

$$J(x,A,r)=\sum {\left(f_{i}-r\right)}^{2}$$

Normalization:

***A*** ← ***A***/|***A***|
***X*** ← (point on axis closest to origin)

Derivatives:

$$\begin{array}{l} \frac{\partial d_{i}}{\partial x}={\left(f_{i}\right)}_{x}\\ \frac{\partial d_{i}}{\partial y}={\left(f_{i}\right)}_{y}\\ \frac{\partial d_{i}}{\partial z}={\left(f_{i}\right)}_{z}\\ \frac{\partial d_{i}}{\partial A}={\left(f_{i}\right)}_{A}\\ \frac{\partial d_{i}}{\partial B}={\left(f_{i}\right)}_{B}\\ \frac{\partial d_{i}}{\partial C}={\left(f_{i}\right)}_{C}\\ \frac{\partial d_{i}}{\partial r}=-1 \end{array}$$

### 3.7 Cone Fitting

Defining parameters:

***x***—a point on the cone axis (not the apex).
***A***—the direction numbers of the cone axis (pointing toward the apex).
*s*—the orthogonal distance from the point, ***x***, to the cone.
*ψ* —the cone’s apex semi-angle.

Distance equation:

$$d(x_{i})=f_{i}\cos\psi +g_{i}\sin\psi -s$$

Objective function:

$$J(x,A,s,\psi )=\sum {\left(f_{i}\cos\psi +g_{i}\sin\psi -s\right)}^{2}$$

Normalization:

***A*** ← ***A***/|***A***|
*x* ← (point on axis closest to origin)
*ψ* ← *ψ* (mod 2π)
if *ψ* > π then [*ψ* ← *ψ* (mod π); ***A*** ← − ***A***]
if $\psi >\frac{\pi }{2}$ then *ψ* ← π – *ψ*
if *s* < 0 then [*s* ← − *s*; ***A*** ← − ***A***]

Derivatives:

$$\begin{array}{l} \frac{\partial d_{i}}{\partial x}={\left(f_{i}\right)}_{x}\cos\psi +{\left(g_{i}\right)}_{x}\sin\psi \\ \frac{\partial d_{i}}{\partial y}={\left(f_{i}\right)}_{y}\cos\psi +{\left(g_{i}\right)}_{y}\sin\psi \\ \frac{\partial d_{i}}{\partial z}={\left(f_{i}\right)}_{z}\cos\psi +{\left(g_{i}\right)}_{z}\sin\psi \\ \frac{\partial d_{i}}{\partial A}={\left(f_{i}\right)}_{A}\cos\psi +{\left(g_{i}\right)}_{A}\sin\psi \\ \frac{\partial d_{i}}{\partial B}={\left(f_{i}\right)}_{x}\cos\psi +{\left(g_{i}\right)}_{x}\sin\psi \\ \frac{\partial d_{i}}{\partial C}={\left(f_{i}\right)}_{x}\cos\psi +{\left(g_{i}\right)}_{x}\sin\psi \\ \frac{\partial d_{i}}{\partial s}=-1\\ \frac{\partial d_{i}}{\partial \psi }=-f_{i}\sin\psi +g_{i}\cos\psi \end{array}$$

### 3.8 Torus Fitting

Defining parameters:

***x***—the torus center.
***A***—the direction numbers of the torus axis.
*r*—the major radius.
*R*—the minor radius.

Distance equation:

$$d(x_{i})=\sqrt{g_{i}^{2}+{\left(f_{i}-r\right)}^{2}-R}$$

Objective function:

$$J(x,A,r,R)=\sum {\left[\sqrt{g_{i}^{2}+{\left(f_{i}-r\right)}^{2}}-R\right]}^{2}$$

Normalization:

*A* ← ***A***/|***A***|

Derivatives:

$$\begin{array}{l} \frac{\partial d_{i}}{\partial x}=\left[g_{i}{\left(g_{i}\right)}_{x}+\left(f_{i}-r\right){\left(f_{i}\right)}_{x}\right]/\left(d_{i}+R\right)\\ \frac{\partial d_{i}}{\partial y}=\left[g_{i}{\left(g_{i}\right)}_{y}+\left(f_{i}-r\right){\left(f_{i}\right)}_{y}\right]/\left(d_{i}+R\right)\\ \frac{\partial d_{i}}{\partial z}=\left[g_{i}{\left(g_{i}\right)}_{z}+\left(f_{i}-r\right){\left(f_{i}\right)}_{z}\right]/\left(d_{i}+R\right)\\ \frac{\partial d_{i}}{\partial A}=\left[g_{i}{\left(g_{i}\right)}_{A}+\left(f_{i}-r\right){\left(f_{i}\right)}_{A}\right]/\left(d_{i}+R\right)\\ \frac{\partial d_{i}}{\partial B}=\left[g_{i}{\left(g_{i}\right)}_{B}+\left(f_{i}-r\right){\left(f_{i}\right)}_{B}\right]/\left(d_{i}+R\right)\\ \frac{\partial d_{i}}{\partial C}=\left[g_{i}{\left(g_{i}\right)}_{C}+\left(f_{i}-r\right){\left(f_{i}\right)}_{C}\right]/\left(d_{i}+R\right)\\ \frac{\partial d_{i}}{\partial r}=-(f_{i}-r)(d_{i}+R)\\ \frac{\partial d_{i}}{\partial R}=-1 \end{array}$$

## 4. Discussion

The algorithms have been implemented in the ATS and have been used for 5 years by NIST and by members of the ASME B89.4.10 Working Group. In general they have performed extremely well. They have successfully solved a number of difficult fitting problems that could not be solved by many commercial software packages used on Coordinate Measuring Systems (CMSs). (Some of the most difficult problems are cylinders or cones sampled over a small patch.) The ATS algorithms have an extremely broad range of convergence. Failure to converge has only been observed for pathological fitting problems (e.g., fitting a circle to collinear points). Special checks can detect most of these situations.

The ATS algorithms are generally robust. For most fits, a good starting guess is not required to reach the global minimum. This is due, in part, to the careful choice of fitting parameters, the use of certain constraints, and, for cylinders and cones, the technique of restarting a search after an initial solution is found.

## Figures and Tables

**Fig. 1:**
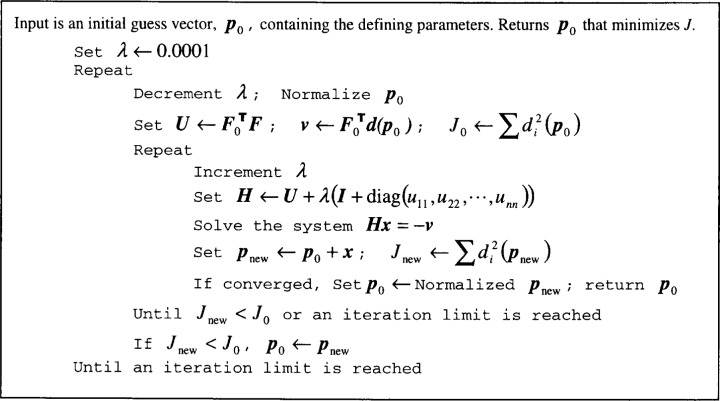
Levenberg-Marquardt algorithm.

## 5. Appendix A. The Levenberg-Marquardt Algorithm

The Levenberg-Marquardt algorithm [11] finds the vector, ***p***, that minimizes an objective function, $J(p)=\Sigma d_{i}^{2}(p)$, where *d_i_*(***p***) is the distance from the point, ***x****_i_*, to the geometry defined by the vector of parameters, ***p***. In the case of a cylinder, for example, ***p*** = (***x***, ***A***, *r*) = (*x*, *y*, *z*, *A*, *B*, *C*, *r*) and *d_i_*(***p***) = *f_i_* − *r*. One derivation of the algorithm [12] begins by approximating *J* as a linear function of ***p***, $\hat{J}(p)$:

$$J(p)\approx \hat{J}(p)=\sum {\left(d_{i}(p_{0})+\nabla d_{i}(p_{0})\cdot p\right)}^{2}$$

where ∇*d_i_*(***p***_0_) is the gradient of *d_i_*(***p***) evaluated at an initial guess, ***p***_0_. This approximation is valid within a certain trust region radius. The derivation then considers how to minimize $\hat{J}(p)$. A search direction is calculated based on $\hat{J}(p)$, and a search is made in that direction within the limits of the trust region radius for a point, ***p***_new_, such that *J*(***p***_new_) < *J*(***p***_0_). When ***p***_new_ is found, it becomes the new ***p***_0_ for another iteration of the above process.

The basis for the algorithm is the result that the solution, ***p****, to each iteration can be expressed as ***p**** = ***p***(λ) = − (***F***_0_**^⊤^*F***_0_ + λ***D*^⊤^*D***)^−1^ ***F***_0_**^⊤^*d***(***p***_0_) where ***F***_0_ is a matrix having ∇*d_i_*(***p***_0_) as its *i*th row, ***D*** is an appropriate weighting matrix, ***d***(***p***_0_) is the vector of residuals, *d_i_*(***p***_0_), and λ ≥ 0 is a variable called the Levenberg-Marquardt parameter. This parameter can be considered the Lagrange multiplier for the constraint that each search be limited to the trust region radius.

The Levenberg-Marquardt algorithm is presented in the figure below. The matrix ***F***_0_**^⊤^*F***_0_ + λ***D*^⊤^*D*** is named ***H*** and the vector ***F***_0_**^⊤^*d***(***p***_0_) is called ***v***. The algorithm includes a modification suggested by Nash [11], which is to use a weighting matrix defined so that ***D*^⊤^*D*** is the identity matrix plus the diagonal of ***F***_0_**^⊤^*F***_0_. Nash’s use of the identity in the definition of ***D*** forces ***H*** to be positive definite. (Note that ***D*** is never calculated.) Since ***H*** is also symmetric, the system ***Hx*** = − ***v*** can be reliably solved using the Cholesky decomposition [9]. We chose 10 and 0.04 as factors with which to increment and decrement λ respectively. Finally, note that we normalize the parameter vector, ***p***, at every iteration. However, normalization of ***p*** never changes the value of the objective function, *J*(***p***). This routine is the basis of the ATS implementation of the Levenberg-Marquardt algorithm.

## 6. Appendix B. Gradient Test for Correctness

One helpful check is to compute the gradient of the objective function at the computed minimum. The correct least-squares solution yields Δ*J* = 0. Also, these derivatives are important because several minimization algorithms require that these derivatives be included. The definitions of *f* and *g* are kept the same as they were in Sec. 3. For every nonlinear geometry, assume the same definition for the objective function, *J*, as specified in Sec. 3. For the linear geometries, the objective functions are here defined in terms of unconstrained parameters.

### Planes

$J(x,A)=\sum g_{i}^{2}$

$\begin{array}{l} \frac{\partial J}{\partial x}=2\sum g_{i}{\left(g_{i}\right)}_{x}\\ \frac{\partial J}{\partial y}=2\sum g_{i}{\left(g_{i}\right)}_{y}\\ \frac{\partial J}{\partial z}=2\sum g_{i}{\left(g_{i}\right)}_{z}\\ \frac{\partial J}{\partial A}=2\sum g_{i}{\left(g_{i}\right)}_{A}\\ \frac{\partial J}{\partial B}=2\sum g_{i}{\left(g_{i}\right)}_{B}\\ \frac{\partial J}{\partial C}=2\sum g_{i}{\left(g_{i}\right)}_{C} \end{array}$

### Lines

$J(x,A)=\sum f_{i}^{2}$

$\begin{array}{l} \frac{\partial J}{\partial x}=2\sum f_{i}{\left(f_{i}\right)}_{x}\\ \frac{\partial J}{\partial y}=2\sum f_{i}{\left(f_{i}\right)}_{y}\\ \frac{\partial J}{\partial z}=2\sum f_{i}{\left(f_{i}\right)}_{z}\\ \frac{\partial J}{\partial A}=2\sum f_{i}{\left(f_{i}\right)}_{A}\\ \frac{\partial J}{\partial B}=2\sum f_{i}{\left(f_{i}\right)}_{B}\\ \frac{\partial J}{\partial C}=2\sum f_{i}{\left(f_{i}\right)}_{C} \end{array}$

### Spheres

$\begin{array}{l} \frac{\partial J}{\partial x}=-2\sum d_{i}(x_{i}-x)/|x_{i}-x|\\ \frac{\partial J}{\partial y}=-2\sum d_{i}(y_{i}-y)/|x_{i}-x|\\ \frac{\partial J}{\partial z}=-2\sum d_{i}(z_{i}-z)/|x_{i}-x|\\ \frac{\partial J}{\partial r}=-2\sum d_{i} \end{array}$

### 2D Circles

$\begin{array}{l} \frac{\partial J}{\partial x}=-2\sum d_{i}(x_{i}-x)/(d_{i}+r)\\ \frac{\partial J}{\partial y}=-2\sum d_{i}(y_{i}-y)/(d_{i}+r)\\ \frac{\partial J}{\partial r}=-2\sum d_{i} \end{array}$

### 3D Circles

$\begin{array}{l} \frac{\partial J}{\partial x}=2\sum \left[g_{i}{\left(g_{i}\right)}_{x}+\left(f_{i}-r\right){\left(f_{i}\right)}_{x}\right]\\ \frac{\partial J}{\partial y}=2\sum \left[g_{i}{\left(g_{i}\right)}_{y}+\left(f_{i}-r\right){\left(f_{i}\right)}_{y}\right]\\ \frac{\partial J}{\partial z}=2\sum \left[g_{i}{\left(g_{i}\right)}_{z}+\left(f_{i}-r\right){\left(f_{i}\right)}_{z}\right]\\ \frac{\partial J}{\partial A}=2\sum \left[g_{i}{\left(g_{i}\right)}_{A}+\left(f_{i}-r\right){\left(f_{i}\right)}_{A}\right]\\ \frac{\partial J}{\partial B}=2\sum \left[g_{i}{\left(g_{i}\right)}_{B}+\left(f_{i}-r\right){\left(f_{i}\right)}_{B}\right]\\ \frac{\partial J}{\partial C}=2\sum \left[g_{i}{\left(g_{i}\right)}_{C}+\left(f_{i}-r\right){\left(f_{i}\right)}_{C}\right]\\ \frac{\partial J}{\partial r}=-2\sum \left(f_{i}-r\right) \end{array}$

### Cylinders

$\begin{array}{l} \frac{\partial J}{\partial x}=2\sum \left(f_{i}-r\right){\left(f_{i}\right)}_{x}\\ \frac{\partial J}{\partial y}=2\sum \left(f_{i}-r\right){\left(f_{i}\right)}_{y}\\ \frac{\partial J}{\partial z}=2\sum \left(f_{i}-r\right){\left(f_{i}\right)}_{z}\\ \frac{\partial J}{\partial A}=2\sum \left(f_{i}-r\right){\left(f_{i}\right)}_{A}\\ \frac{\partial J}{\partial B}=2\sum \left(f_{i}-r\right){\left(f_{i}\right)}_{B}\\ \frac{\partial J}{\partial C}=2\sum \left(f_{i}-r\right){\left(f_{i}\right)}_{C}\\ \frac{\partial J}{\partial r}=-2\sum \left(f_{i}-r\right) \end{array}$

### Cones

$\begin{array}{l} \frac{\partial J}{\partial x}=2\sum d_{i}\left[{\left(f_{i}\right)}_{x}\cos\psi +{\left(g_{i}\right)}_{x}\sin\psi \right]\\ \frac{\partial J}{\partial y}=2\sum d_{i}\left[{\left(f_{i}\right)}_{y}\cos\psi +{\left(g_{i}\right)}_{y}\sin\psi \right]\\ \frac{\partial J}{\partial z}=2\sum d_{i}\left[{\left(f_{i}\right)}_{z}\cos\psi +{\left(g_{i}\right)}_{z}\sin\psi \right]\\ \frac{\partial J}{\partial A}=2\sum d_{i}\left[{\left(f_{i}\right)}_{A}\cos\psi +{\left(g_{i}\right)}_{A}\sin\psi \right]\\ \frac{\partial J}{\partial B}=2\sum d_{i}\left[{\left(f_{i}\right)}_{B}\cos\psi +{\left(g_{i}\right)}_{B}\sin\psi \right]\\ \frac{\partial J}{\partial C}=2\sum d_{i}\left[{\left(f_{i}\right)}_{C}\cos\psi +{\left(g_{i}\right)}_{C}\sin\psi \right]\\ \frac{\partial J}{\partial \psi }=2\sum d_{i}\left[-f_{i}\sin\psi +g_{i}\cos\psi \right]\\ \frac{\partial J}{\partial s}=-2\sum d_{i} \end{array}$

### Tori

$\begin{array}{l} \frac{\partial J}{\partial x}=2\sum \frac{d_{i}}{d_{i}+R}\left[g_{i}{\left(g_{i}\right)}_{x}+\left(f_{i}-r\right){\left(f_{i}\right)}_{x}\right]\\ \frac{\partial J}{\partial y}=2\sum \frac{d_{i}}{d_{i}+R}\left[g_{i}{\left(g_{i}\right)}_{y}+\left(f_{i}-r\right){\left(f_{i}\right)}_{y}\right]\\ \frac{\partial J}{\partial z}=2\sum \frac{d_{i}}{d_{i}+R}\left[g_{i}{\left(g_{i}\right)}_{z}+\left(f_{i}-r\right){\left(f_{i}\right)}_{z}\right]\\ \frac{\partial J}{\partial A}=2\sum \frac{d_{i}}{d_{i}+R}\left[g_{i}{\left(g_{i}\right)}_{A}+\left(f_{i}-r\right){\left(f_{i}\right)}_{A}\right]\\ \frac{\partial J}{\partial B}=2\sum \frac{d_{i}}{d_{i}+R}\left[g_{i}{\left(g_{i}\right)}_{B}+\left(f_{i}-r\right){\left(f_{i}\right)}_{B}\right]\\ \frac{\partial J}{\partial C}=2\sum \frac{d_{i}}{d_{i}+R}\left[g_{i}{\left(g_{i}\right)}_{C}+\left(f_{i}-r\right){\left(f_{i}\right)}_{C}\right]\\ \frac{\partial J}{\partial r}=2\sum \frac{d_{i}}{d_{i}+R}\left[g_{i}{\left(g_{i}\right)}_{r}-\left(f_{i}-r\right)\right]\\ \frac{\partial J}{\partial R}=-2\sum d_{i} \end{array}$
